# Antioxidant Enzymes and Heat Shock Protein Genes from *Liposcelis bostrychophila* Are Involved in Stress Defense upon Heat Shock

**DOI:** 10.3390/insects11120839

**Published:** 2020-11-27

**Authors:** Ze Qing Miao, Yan Qing Tu, Peng Yu Guo, Wang He, Tian Xing Jing, Jin Jun Wang, Dan Dan Wei

**Affiliations:** 1 Key Laboratory of Entomology and Pest Control Engineering, College of Plant Protection, Southwest University, Chongqing 400715, China; m15310403421@email.swu.edu.cn (Z.Q.M.); tt961210@email.swu.edu.cn (Y.Q.T.); guopy736498@email.swu.edu.cn (P.Y.G.); hewang1222@email.swu.edu.cn (W.H.); jingtx@yzu.edu.cn (T.X.J.); wangjinjun@swu.edu.cn (J.J.W.); 2 Academy of Agricultural Sciences, Southwest University, Chongqing 400715, China

**Keywords:** psocids, stored product pest, oxidative stress, heat treatment, expression profile

## Abstract

**Simple Summary:**

*Liposcelis bostrychophila* is one of the most serious pests of stored commodities among the psocids. Controlling psocids mainly relies on chemical insecticides and heat stress. In fact, *L. bostrychophila* has developed high levels of resistance or tolerance to heat treatment in grain storage systems. In this study, we evaluated the changes in malondialdehyde (MDA) concentration after different high temperatures. The result showed that MDA is increased slightly overall, but a drastic increase is detected at 42.5 °C for exposure of different times. To further explore the principles of *L. bostrychophila* in response to heat stress, we tested the changes of superoxide dismutase (SOD), catalase (CAT), peroxidases (POD) and glutathione-S-transferases (GST) activities under different heat treatments and identified four inducible *LbHsp70* genes and one *LbHsp110* gene. Enzyme activities and transcript levels changed drastically after different heat treatments. These findings contribute to our understanding of the mechanism of *L. bostrychophila* responding to heat stress and provide baseline information for further understanding the excellent targets of *L. bostrychophila*.

**Abstract:**

Psocids are a new risk for global food security and safety because they are significant worldwide pests of stored products. Among these psocids, *Liposcelis bostrychophila* has developed high levels of resistance or tolerance to heat treatment in grain storage systems, and thus has led to investigation of molecular mechanisms underlying heat tolerance in this pest. In this study, the time-related effects of thermal stress treatments at relatively high temperatures on the activities of antioxidant enzymes, including superoxide dismutase (SOD), catalase (CAT), peroxidases (POD), glutathione-S-transferases (GST) and malondialdehyde (MDA), of *L. bostrychophila* were determined. Thermal stress resulted that *L. bostrychophila* had a significantly higher MDA concentration at 42.5 °C, which indicated that the heat stress increased lipid peroxidation (LPO) contents and oxidative stress in this psocid pest. Heat stress also resulted in significant elevation of SOD, CAT and GST activities but decreased POD activity. Our data indicates that different antioxidant enzymes contribute to defense mechanisms, counteracting oxidative damage in varying levels. POD play minor roles in scavenging deleterious LPO, while enhanced SOD, CAT and GST activities in response to thermal stress likely play a more important role against oxidative damage. Here, we firstly identified five *LbHsps* (four *LbHsp70s* and one *LbHsp110*) from psocids, and most of these *LbHsps* (except *LbHsp70-1*) are highly expressed at fourth instar nymph and adults, and *LbHsp70-1* likely presents as a cognate form of HSP due to its non-significant changes of expression. Most *LbHsp70s* (except *LbHsp70-4*) are significantly induced at moderate high temperatures (<40 °C) and decreased at extreme high temperatures (40–45 °C), but *LbHsp110-1* can be significantly induced at all high temperatures. Results of this study suggest that the *LbHsp70s* and *LbHsp110* genes are involved in tolerance to thermal stress in *L. bostrychophila*, and antioxidant enzymes and heat shock proteins may be coordinately involved in the tolerance to thermal stress in psocids.

## 1. Introduction

Temperature is one of the most important environmental factors that affect life history, behavioral and physiological traits and community composition in insects [1]. As heterothermic animals, insects have evolved a variety of behavioral and physiological adaptations to avoid temperature impairments [2,3]. Thermal stress tolerance is essential for completion of insect life cycles, and insects may exhibit alterations in behavior, morphology, life history and physiological characteristics when exposed to thermal stress. Enhanced antioxidant enzymes activities and the production of heat shock proteins (HSPs) have been widely considered to be the most important strategies that help insects generate tolerance to temperature stress [4,5,6,7].

Thermal stress can lead to oxidative damage and oxidative stress, which involves elevated intracellular levels of reactive oxygen species (ROS) that can damage lipids, proteins and DNA [8]. Avoiding ROS damage, antioxidant enzymes are involved in the oxidative damage response [9,10]. Generally, there is a balance between ROS production and antioxidant processes. However, thermal stress can disrupt this balance and lead to synthesis of additional ROS, and thus result in LPO via cell lipids disruption [11,12]. LPO could be determined indirectly by measuring malondialdehyde (MDA) concentration [13]. In insects, the primary antioxidative enzymes mainly include superoxide dismutase (SOD), catalase (CAT), peroxidase (POD) and glutathione S-transferases (GSTs) [14,15,16]. SOD converts superoxide anion (O_2_^−^) into oxygen (O_2_) and hydrogen peroxide (H_2_O_2_), while CAT and POD break down H_2_O_2_ into H_2_O and O_2_. GST removes the harmful products of LPO or hydroperoxides from cells [13,15]. The remarkable increase of the antioxidant enzymes’ activities is a sign of oxidative stress, as well as an indicator of strong capability for combating oxidative stress through eliminating ROS in cells [1,17].

Heat shock proteins are highly conserved proteins that are synthesized as stress proteins when an organism is exposed to stresses, e.g., extreme temperatures, disease, toxins, hypoxia, ionizing and ultraviolet (UV) radiations. HSPs are a first line of defense against stressful damage, as well as playing important roles in molecular and physiological functions, i.e., DNA repair, oogenesis, embryo development and diapause [18]. According to their molecular weights and sequence similarities, HSPs can be divided into five families, including small Hsps (sHsps), Hsp60s, Hsp70s, Hsp90s and Hsp110s [19]. Among these HSPs families, Hsp70s is one of the most heat-inducible and evolutionally conserved proteins in terms of structure and function [20]. Hsp70 may also prevent apoptosis by interfering with the signaling events that trigger the apoptotic process [21]. Two major conserved domains are usually associated with Hsp70 function, i.e., nucleotide-binding domain (NBD) and substrate-binding domain (SBD) [22].

Hsp110s have close relationships with Hsp70s, but they generally exhibit a longer C-terminal extension. Hsp110s are essentially described as chaperones preventing the aggregation of denatured proteins [23]. *Hsp110* genes act as a nucleotide exchange factor, releasing peptide substrate from Hsp70 in an ATP-dependent manner. Recently, a study showed that *Hsp110* genes can be induced by heat stress in animals, and moreover, they showed different responses to thermal stress in different species from the same genus [24]. *Hsp110* genes may be heat-inducible only in the more thermo-tolerant species, and this trait allows the differential expansion of one species relative to another closely related species under current climate change scenarios. *Hsp110s* are sometimes still mentioned as a subgroup of the Hsp70 family due to their function as a nucleotide exchange factor for Hsp70, often in complex with the co-chaperone Hsp40. However, recent structural studies suggest that they belong to different families because of their functional differences with *Hsp70s* [18].

The genus *Liposcelis* psocids, also named booklice, is a group of important stored-product pests worldwide, and *L. bostrychophila* is one of the most serious pests of stored commodities among the psocids [25]. Recently, *L. bostrychophila* and *L. entomophila* has developed high levels of resistance or tolerance to heat treatment in grain storage systems [26,27]. Meanwhile, different *Liposcelis* species and development stages usually differ in heat tolerance. For example, the egg stage is the most tolerant (heating to 46 °C for >35 h can control eggs of *L. bostrychophila*). In contrast, *L. decolor* is the most tolerant species at 46–51 °C and *L. paeta* is most tolerant below 46 °C, but most susceptible at 47–51 °C [28]. Presently, the physiological and molecular mechanisms behind the thermal tolerance in psocids is not fully understood. One study showed that differences in heat tolerance may be due partly to physiological protection afforded by the generation of small heat-inducible proteins, HIP23 and HIP27 in *L. entomophila* [27].

In this study, the time-related effects (durations of 2, 4 and 8 h) of thermal stress treatments of *L. bostrychophila* at comparatively high (37.5, 40, 42.5 and 45 °C) temperatures on the activities of antioxidant enzymes, including SOD, CAT, GST, POD as well as LPO levels (MDA), were determined. Besides the antioxidant enzymes, the expression profiles of five *LbHsps* under different heat stress (35, 37.5, 40, 42.5 and 45 °C) and development stages were also investigated. The aim of this study was to determine how variations in temperature affect antioxidant enzyme activities and HSPs in response to oxidative stress in psocids. Such changes may explain why *L. bostrychophila* has a very high-temperature plasticity. Studies like this can provide new insights into the molecular mechanisms underlying heat tolerance in psocids and novel avenues for promoting the application of heat treatments of this pest.

## 2. Materials and Methods

### 2.1. Insect Rearing

The laboratory colony of *L. bostrychophila* was originally collected from grain storage facilities in Beibei, Chongqing, China, in 2002. *L. bostrychophila* was reared with an artificial diet consisting of whole-wheat flour, yeast powder and skimmed milk (10:1:1) in and incubator at 27 ± 0.5 °C and 75–80% relative humidity with a scotophase of 24 h.

### 2.2. Insect Collection, Thermal Stress and Protein Extraction

The sampling of different developmental stages was performed according to Wang’s method [26]. Based on the life history of *L. bostrychophila* at 27.5 °C, 5, 11, 15, 19, 22 and 26 days were used for collecting the eggs, first-, second-, third- and fourth-stadium nymphs, and adults, respectively. All the insects were collected under a microscope using soft brushes, and collected samples were stored at −80 °C for future use.

For antioxidant enzyme assays and *LbHsps* expression analysis, adults weighing approximately 0.1 g were transferred into plastic dishes. Four dishes with psocids were exposed at each target temperature including 37.5, 40, 42.5 and 45 °C. The duration of each temperature treatment was 2, 4 and 8 h for antioxidant enzyme assays. Psocids kept at a temperature of 27.5 °C served as a control. Because the measurements of each assay for the control (27.5 °C) did not vary significantly in a very short term (<8 h), the data for the control were pooled as one in the analysis. To evaluate the expression levels of *LbHsps* under different high-temperature treatments, one hundred newly emerged adults were exposed at a target temperature for 1 h. The treatments were achieved by using a programmable temperature controller (Panasonic MIR-154-PC, Kadoma, Osaka, Japan). After treatments, the living adults were frozen immediately in liquid nitrogen and stored at −80 °C until assay. Each treatment was replicated three times. The treated adults were first homogenized in 120 mL ice-cold 0.05 M pH 7.0 phosphate buffers. The crude homogenates were centrifuged at 4 °C and 10,000× *g* for 10 min. The supernatants were collected and stored on ice until assayed. The protein concentration was determined using the Bradford method [29], with bovine serum albumin as standard.

### 2.3. Enzyme Activity and MDA Assay

The activity of SOD (Item number: A001-3-2), CAT (Item number: A007-1-1), POD (Item number: A084-1-1), GST (Item number: A004) and MDA (Item number: A003-1-2) was determined using commercial assay kits (Nanjing Jiancheng Bioengineering Institute, Jiangsu, China) following the manufacturer’s instructions, with some slight modifications. The absorbance was read in a microplate spectrophotometer (XMark™, Bio-Rad, Hercules, CA, USA). MDA concentration and enzyme activities were calculated according to the methods that have been reported in *H. graminicola* [30].

### 2.4. RNA Isolation and cDNA Cloning of LbHsps

Total RNA was extracted using TRIzol Reagent (Invitrogen Life Technologies, Carlsbad, CA, USA) according to the manufacturer’s protocols. RNA quality, purity and concentration were evaluated by measuring the absorbance at OD_260/280_ using a NanoVue spectrophotometer (GE Healthcare Biosciences, Uppsala, Sweden). The cDNA for cloning and qPCR was synthesized using 1 μg of total RNA with the PrimeScript^®^ RT reagent Kit (Takara Biotechnology Dalian Co., Ltd., Dalian, China) following the manufacturer’s protocol.

Five heat shock protein genes were identified based on the *L. bostrychophila* transcriptome database [31]. The transcripts, which contain the full-length open reading frames (ORFs), were verified by PCRs with specific primers (Table 1). PCRs were conducted using PrimeSTAR high-fidelity DNA polymerase (Takara, Dalian, China), in accordance with the following protocol: 95 °C for 3 min, followed by 34 cycles of 95 °C for 30 s, 60 °C for 30 s, and 72 °C for 2 min and then with a final extension at 72 °C for 10 min. The amplified products were purified by electrophoresis on agarose gels (1.0–1.5%) and cloned into the pGEM-T Easy vector (Promega, Fitchburg, MA, USA). After confirmation by PCR using primers M13F and M13R, the positive clones containing target genes were isolated and sequenced (BGI, Beijing, China).

### 2.5. Gene Characterization and Phylogenetic Analysis

Sequence alignments were carried out using DNAMAN software (DNAMAN 5.2.2, Lynnon BioSoft, Quebec, QC, Canada), and ORFs were identified using the ORF Finder (https://www.ncbi.nlm.nih.gov/orffinder/). The molecular weight and isoelectric point of the amino acid sequences were computed by ExPASy Server (http://www.expasy.org/tools/protparam.html). The conserved motifs presented in deduced amino acid sequences were identified using InterPro (http://www.ebi.ac.uk/interpro/scan.html) or BLASTP in NCBI. A phylogenetic tree based on the amino acid sequences of LbHSPs and other insect HSPs was constructed with MEGA 7.0 software using the neighbor-joining method, and bootstrap values were calculated on 1000 replications [32].

### 2.6. Quantitative Real-Time PCR (qPCR)

The expression profiles of the target genes at different developmental stages and high-temperature exposures were conducted by qPCR. All primers for qPCR were designed in Primer 3.0 (http://frodo.wi.mit.edu/) (Table 1). The *β*-actin gene (FJ447483) was used to normalize results of target gene expression and to correct for sample-to-sample variation. qPCR was carried out in a Mx3000P thermal cycler (Stratagene, La Jolla, CA, USA) with 20 µL reaction volumes containing 1 µL of template cDNA, 10 µL Go Taq^®^ qPCR Master Mix, 1 µL each of forward and reverse primers (0.2 mM), 0.2 µL Rox and 6.8 µL ddH_2_O. Thermal cycling conditions were: 95 °C for 2 min, followed by 40 cycles of at 95 °C for 15 s and 60 °C for 30 s. A final melt-curve step (ramping from 60 to 95 °C in 0.6 °C steps every 5 s) at the end was conducted to ensure the consistency and specificity of the amplified product. All of these experiments involved three biological replications. Relative gene expression data were calculated according to the 2^–ΔΔCT^ method [33]. All qPCR assays were validated in compliance with the Minimum Information for Publication of Quantitative Real-Time PCR Experiments (MIQE) guidelines [34].

### 2.7. Statistical Analysis

Differences in antioxidant enzyme activity assays and *LbHsps* expression levels among different developmental stages and high-temperature treatments were analyzed by two-way (temperature and treatment duration) or one-way analysis of variance (ANOVA) followed by Tukey’s tests in SPSS 19.0 (SPSS, Inc., Chicago, IL, USA). All the data were expressed as mean ± standard error (SE), and the level of significance was set at *p* < 0.05.

## 3. Results

### 3.1. Changes in MDA

The effects of different heat stresses on MDA concentration of *L. bostrychophila* are presented in Table 2. MDA concentrations in *L. bostrychophila* were significantly affected by treatment temperatures (*F*_4,44_ = 366.523; *p* < 0.001) and durations (*F*_2,44_ = 115.127; *p* < 0.001). The results showed that the highest concentration of MDA with increasing temperature was formed at 42.5 °C (7.763 nmol·mg^−1^ protein for 2 h, 17.505 nmol·mg^−1^ protein for 4 h and 5.720 nmol·mg^−1^ protein for 8 h). When heat stress lasted 4 h, the concentration of MDA was significantly increased with increasing temperatures. However, for exposure of 2 and 8 h, MDA concentration was not significantly enhanced (except at 42.5 °C) compared to controls.

### 3.2. Antioxidant Enzyme Activities

**Figure 1:**
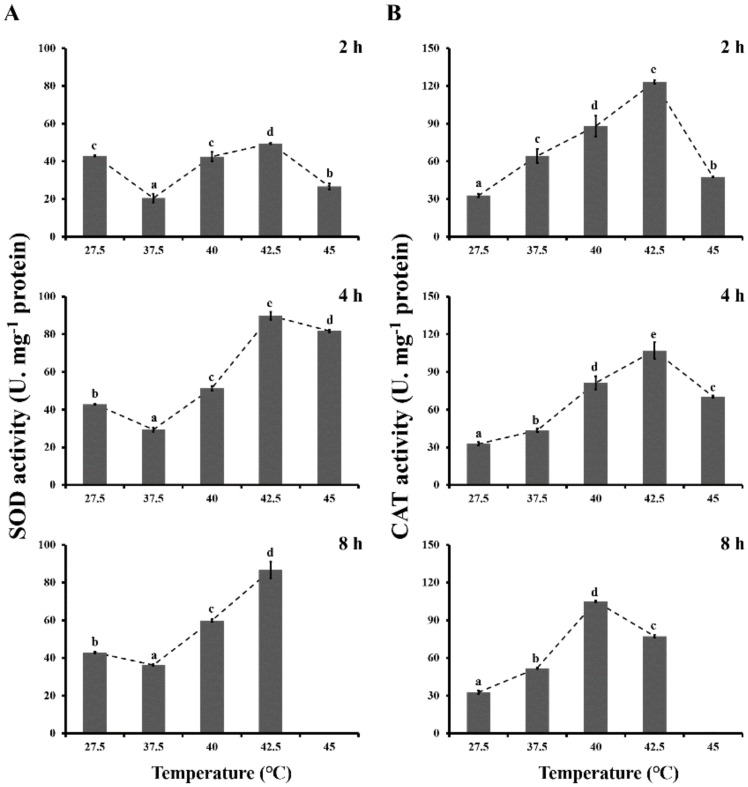
Effects of different thermal stresses on superoxide dismutase (SOD) and catalase (CAT) activities (U·mg^−1^ protein) in *Liposcelis bostrychophila*. (**A**) SOD activity. (**B**) CAT activity. The temperature of 27.5 °C served as a control. Each value represents the mean (±SE) of three replications. Different letters topped on the bar designated significant difference at *p* < 0.05. No data was available at 45 °C of 8 h exposure due to the death of tested insects.

The SOD activities of *L. bostrychophila* under heat thermal stresses are presented in Figure 1A. SOD activities were significantly affected by exposure to high-temperature treatments (*F*_4,44_ = 338.253; *p* < 0.001) and various durations (*F*_2, 44_ = 228.889; *p* < 0.001). In some high-temperature treatments (40 and 42.5 °C), SOD activities gradually increased as the temperature increased for durations of 4 and 8 h when compared with the control (27.5 °C). Meanwhile, the highest activity was found at 42.5 °C (49.456 U·mg^−1^ protein for 2 h, 89.805 U·mg^−1^ protein for 4 h and 86.716 U·mg^−1^ protein for 8 h). However, SOD activities decreased significantly at 37.5 °C in all treatment durations. 

Changes in CAT activities of *L. bostrychophila* after the heat shock for different durations are presented in Figure 1B. CAT activities were significantly affected by the high temperatures (*F*_4,44_ = 235.305; *p* < 0.001) and durations (*F*_2,44_ = 34.430; *p* < 0.001). Compared to control (27.5 °C), a significant upregulation of CAT activities was detected during all heat shock treatments for three durations (2, 4 and 8 h). When exposure lasted 2 and 4 h, CAT activities reached a maximum at 42.5 °C (123.096 U·mg^−1^ protein for 2 h, 106.972 U·mg^−1^ protein for 4 h), whereas the highest activity was found at 40 °C for exposure of 8 h (105.010 U·mg^−1^ protein).

**Figure 2:**
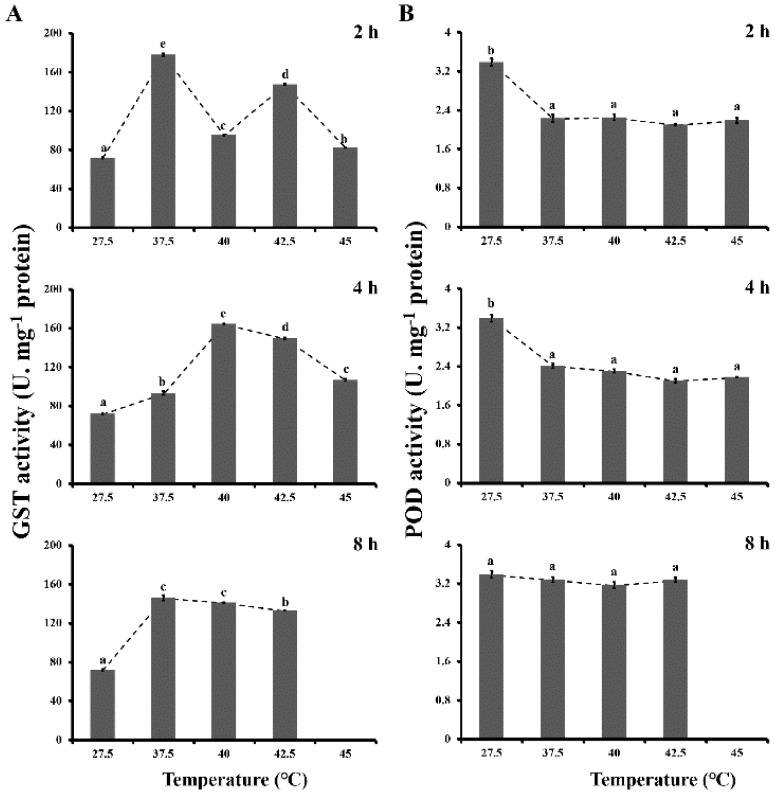
Effects of different thermal stresses on glutathione-S-transferases (GST) and peroxidases (POD) activities (U·mg^−1^ protein) in *Liposcelis bostrychophila*. (**A**) GST activity. (**B**) POD activity. The temperature of 27.5 °C served as a control. Each value represents the mean (±SE) of three replications. Different letters topped on the bar designated significant difference at *p* < 0.05. No data was available at 45 °C of 8 h exposure due to the death of tested insects.

The effects of different heat stress on GST activities of *L. bostrychophila* are presented in Figure 2A. The statistical analysis showed that GST activities were significantly affected by treatment temperatures (*F*_4,44_ = 3393.354; *p* < 0.001) and durations (*F*_2,44_ = 381.410; *p* < 0.001). GST activities increased significantly during all heat shock treatments for three durations (2, 4 and 8 h) in comparison with the control (27.5 °C). The highest activities were found for exposure of 2 and 8 h at 37.5 °C and for exposure of 4 h at 40 °C (177.715 U·mg^−1^ protein for 2 h, 164.335 U·mg^−1^ protein for 4 h and 146.171 U·mg^−1^ protein for 8 h). GST activities increased in various degrees for 2 h duration by 147.5% at 37.5 °C, 32.3% at 40 °C, 105.0% at 42.5 °C and 14.6% at 45 °C respectively, when compared with the control.

POD activities of *L. bostrychophila* under different thermal stress are presented in Figure 2B. POD activity was found to have a significant difference among the different treatment temperatures (*F*_4,44_ = 479.776; *p* < 0.001) and durations (*F*_2,44_ = 15.803; *p* < 0.001). However, for exposure of 2 and 4 h heat shock treatments, the POD activities were significantly decreased compared to the control, but there was no obvious change among themselves.

### 3.3. Sequence Analysis and Phylogenetic Tree

**Figure 3:**
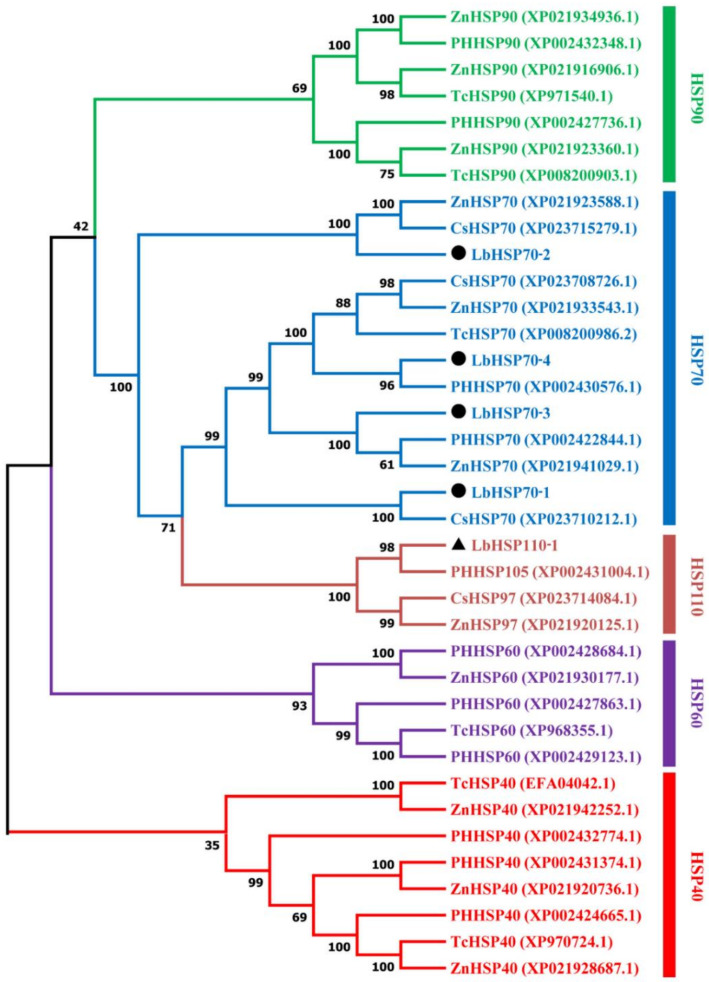
Neighbor-joining phylogenetic tree of *LbHsps* with other insects. The *LbHsp70s* are labeled with a black circle and *LbHsp110-1* with a black triangle. Numbers on the branches are the bootstrap values obtained from 1000 replicates. Sequences were downloaded from NCBI. The GenBank accession numbers are shown after the sequence names. The abbreviation of different insect species: Cs: *Cryptotermes secundus*, PH: *Pediculus humanus corporis*, Tc: *Tribolium castaneum*, Zn: *Zootermopsis nevadensis*.

In this study, four genes encoding Hsp70s and one gene encoding Hsp110 were identified in *L. bostrychophila*, namely, *LbHsp70-1*, *LbHsp70-2*, *LbHsp70-3*, *LbHsp70-4* and *LbHsp110-1*. For four *LbHsp70* genes, the length of the deduced amino acid sequences varied from 505 to 690 amino acids, and the predicted molecular weight ranged from 125.2 to 167.4 kDa with the theoretical isoelectric points of 4.95–5.06. For *LbHsp110* gene, the length of the deduced amino acid sequence is 829 amino acids, and the predicted molecular weight is 201.3 kDa with the theoretical isoelectric point of 4.91 (Supplementary Table S1). Multiple alignments of amino acid sequences of *LbHsps* were conducted (Supplementary Figures S1 and S2). The similarity of amino acid sequences among four *LbHsp70* genes is 50.90%, and the similarity of amino acid sequences between *LbHsp110* gene and four *LbHsp70* genes is 38.77%. The deduced amino acid sequences of these genes contained signature sequences and motifs typical of the Hsp70 family and Hsp110 family (Supplementary Figures S1 and S2). The C-termini of three *LbHsp70s* contain the conserved EEVD motif, which enables Hsp70 to interact with chaperones in the cytoplasm [35]. The N-termini of the *LbHsp70s* contain a conserved sequence with an ATP-GTP binding site, which is associated with conformational switching and ATPase activity. The constructed phylogeny tree showed that five *LbHsp* genes fall into two clades (Figure 3). Phylogenetic analysis with other well-documented HSP proteins clearly classified *LbHsp70-1*, *LbHsp70-2*, *LbHsp70-3* and *LbHsp70-4* into the Hsp70 clade, and *LbHsp110-1* was located in the Hsp110 clade.

### 3.4. Expression Profiling of Five LbHsps

**Figure 4:**
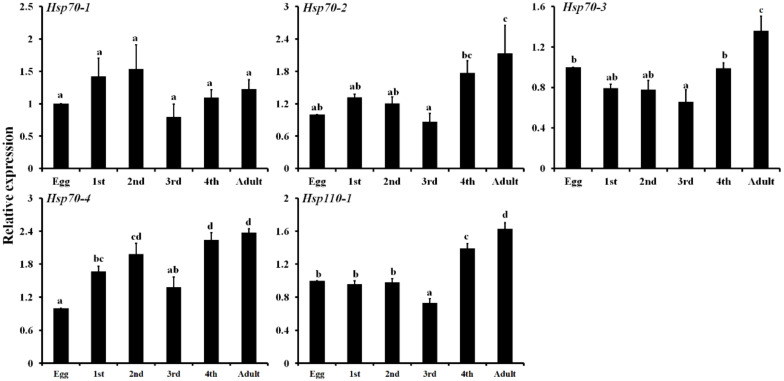
Developmental expression pattern of the five *Hsps* genes in *Liposcelis bostrychophila*. Each value represents the mean (±SE) of three replications. Different letters above the error bar for each gene show significant difference (*p* < 0.05).

**Figure 5:**
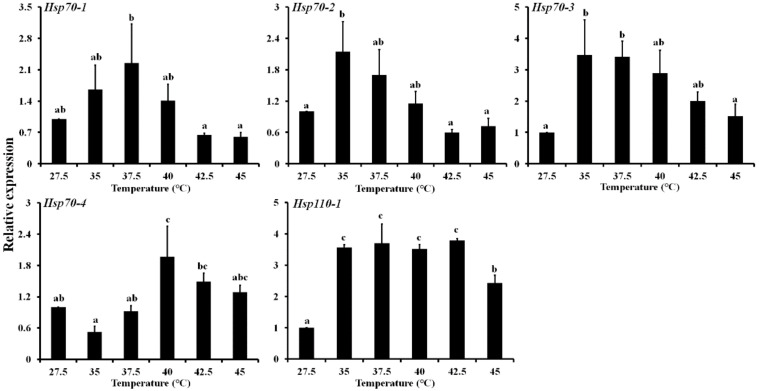
Different thermal stresses induction on the expression profiles of the five *Hsps* genes in *Liposcelis bostrychophila*. Bar graph presents values as mean (±SE) of three replications. Different letters above the error bar for each gene show significant difference (*p* < 0.05).

The expression patterns of *LbHsps* at different development stages (eggs, first to fourth-instar nymph, adults) were investigated by qPCR. The results showed that all five genes were consistently expressed for all the tested stages, and expression of these genes varied in various developmental stages (Figure 4). All the genes were highly expressed in adults except the *LbHsp70-1*. Generally, the relative expressions of five *LbHsp* genes in the egg and the third nymph stage are lower than that in other stages. For *LbHsp70-4*, the transcriptional level of the three nymph (first, second, and fourth) stages and adult stages were significantly higher than that in the egg stage, with an increase of 1.67-, 1.99-, 2.24- and 2.37-fold, respectively. The mRNA abundances of the five *LbHsps* were also evaluated in response to high-temperature stresses by qPCR (Figure 5). These *LbHsp* genes were upregulated in different degrees by heat treatments below 42.5 °C, and then the abundances of these genes declined at higher temperatures. For *LbHsp110-1*, the mRNA expression levels were significantly increased by 3.57-, 3.70-, 3.51-, 3.79- and 2.42-fold respectively, in comparison with the control (Figure 5).

## 4. Discussion

Many studies of insect–thermal stress interaction have revealed that insects have evolved complex protective mechanisms to protect themselves against high temperatures, and antioxidant enzymes and heat shock proteins are the most well-known effectors in this process [6,9]. Insects are usually equipped with a comprehensive antioxidant defense system to relieve oxidative stress and alleviate the effect of damaged macromolecules produced by thermal stress.

MDA is a biological marker of oxidative stress. MDA can be used to determine the degree of lipid peroxidation, because it is a major oxidation product of peroxidized polyunsaturated fatty acids [36,37]. In the current study, MDA concentration is increased slightly overall, but a drastic increase is detected at 42.5 °C for exposure of different times. These results indicated that high-temperature stress led to increased damage of lipids by ROS in this psocid. The normal level of MDA might be due to enhanced activities of antioxidative enzymes or other defense pathways, which can scavenge excess lipid peroxidation. This study demonstrated that in *L. bostrychophila*, higher temperature stress was accompanied by lipid peroxidation and other responses to oxidative stress, and the similar results were also reported in *P. japonica* and *H. graminicola* [1,30].

SOD plays an important role in antioxidant defense against ROS, reducing levels of superoxide radical, which is induced by high temperatures [38]. In this study, a significant increase of SOD activities was observed in *L. bostrychophila* adults exposed to high temperature (>37.5 °C) at 4 and 8 h, and it reached maximum values at 42.5 °C. Moreover, SOD activities increase gradually along with the prolongation of exposure time. These results suggest that SOD activity might be an adaptive response of psocids to overcome high-temperature-induced superoxide anion toxicity. Our results are partially consistent with the findings in *P. japonica, M. separata, B. dorsalis* and *N. cucumeris* exposed to heat stress [1,39,40,41]. Thus, we infer that SOD plays an important role in the response to short-term and long-term phasic thermal stress in *L. bostrychophila*.

CAT is the principal H_2_O_2_ scavenging enzyme, and SOD and CAT work together in stepwise oxygen reduction [42], but CAT removes H_2_O_2_ only at high concentrations and has little effect at low concentrations [43]. In this study, CAT activities in *L. bostrychophila* adults are higher than controls under different high-temperature stress. Interestingly, with the increase of exposure temperature, CAT activities reach the highest activity at 40 or 42.5 °C, and then reduced slightly at 42.5 or 45 °C. The reasons for these changes of CAT activities are unclear, but may be associated with changes of SOD activities. The similar phenomenon was also found in some previous studies of *P. japonica* and *B. dorsalis* [1,17].

GST is a group of multifunctional dimer enzymes that can catalyze the combination of glutathione and a variety of endogenous and heterologous compounds to discharge toxins. It has the function of protecting cells from oxidative damage, isomerization and intercellular transport [44]. The elevated GST activities suggest that GST in *L. bostrychophila* is involved in the inactivation of toxic lipid peroxidation products accumulated due to oxidative damage by heat shock stress. Similar findings are also reported in *P. japonica*, *P. citri*, *M. separate*, *C. suppressalis* and *A. mylitta* [1,9,39,42,45]. H_2_O_2_ is also broken down by POD, which can metabolize lipid peroxidation products together with GST [17]. In this study, the POD activities exhibited varied decreased changes under different high-temperature stress at 2 and 4 h exposures. We speculate that other peroxidase enzymes, i.e., CAT, may remove most of these hydrogen peroxides produced by SOD, and thus CAT and GSTs may play a more important role in removing excess ROS than POD when *L. bostrychophila* is exposed to extreme temperature stress.

Previous studies demonstrated that HSPs play important roles in thermal tolerance of insects [19]. Generally, *Hsp* genes are developmentally regulated in insects, and the role of HSPs in the regulation of insect growth and development has become an important area of inquiry. In fact, Hsps act within networks, and the functions of individual proteins within an Hsp family often differ with developmental stages, subcellular location and environmental conditions. Here, we show that five *LbHsps* have different expression levels in different developmental stages. These *LbHsp* genes are usually highly expressed in fourth nymph and adults, whereas their expressions are lowest at the third nymph stage. Other studies also showed that Hsp70s genes could participate in the regulation of growth and development in some insects [46,47]. For example, the expression of *Hsp70* III from *T. castaneum* varied among developmental stages, suggesting that *TcHsp70* III is developmentally regulated [48]. Therefore, we inferred that these Hsp proteins may participate in the development process of *L. bostrychophila,* and the function of these Hsps may be associated with the ability of defense against stress in the life history of this pest.

Many studies have shown that genes encoding Hsp70 and Hsp20 are generally more susceptible to thermal stress than other Hsps [20]. Generally, the Hsp70 genes were expressed at low levels in normal conditions but were induced differently in response to heat stress [49]. The expression of *LbHsp70-4* was significantly increased only at 40 °C, but the other *LbHsp70s* were significantly induced at moderate high temperature (<40 °C) and decreased at extreme high temperature (40–45 °C). Consistent with this result, in *Sitodiplosis mosellana*, the expression of *SmosHsp70* first increased and then decreased with increasing temperature treatment [50]. These similar expression patterns of Hsp90, Hsp70, Hsp60 and small Hsps were also observed in *A. gifuensis*, *M. cinxia* and *F. occidentalis* [6,49,51]. It should be noted that *LbHsp110-1* can be significantly induced at all high temperatures tested (Figure 5). To date, relatively little is known about the expression and the physiological function of insect HSP110s, while numerous studies of Hsp110 have been conducted in mammals and yeast. Previous studies showed that Hsp110 members are co-chaperones of mammalian and yeast Hsp70 chaperones and act as nucleotide exchange factors (NEF) during the ATP hydrolysis cycle [52,53]. Recent studies also showed that Hsp110 genes can be induced by heat stress and play important roles in animal reproduction [24,54].

Differential heat shock tolerance among the stored product psocids *Liposcelis* species was earlier reported [27,28]. In a previous study, *L. entomophila* possesses higher relative tolerance to heat shock stress compared to *L. reticulatus* because it expresses two small Hsps (i.e., HIP 23 and HIP 27). However, the expression of Hsp70 was not observed under heat stress in the above psocids [27]. The reasons for non-detection of Hsp70 expression in the above-mentioned psocids by using Western blot analysis is mainly due to the insensitive technology using a monoclonal antibody or possible breakdown of Hsp70. Combined with the former research results, we conclude that Hsp110 and sHsps may greatly help *Liposcelis* psocids to tolerate heat, and thus lead to evolution of heat resistance in this pest. We also tentatively infer that *LbHsp70s* play an important role in *L. bostrychophila* adults in adapting to thermal stress. The current study suggests that heat tolerance in psocids might lead to their more common occurrence in grain stored worldwide, and the response to heat tolerance in *L. bostrychophila* might facilitate its development of thermal resistance to heat treatments.

## 5. Conclusions

The present study confirmed that thermal stress disturbs the redox balance in *L. bostrychophila*, which leads to oxidative stress. To overcome this stress, antioxidant enzymes, i.e., SOD, CAT and GST, and heat shock proteins are involved in antioxidant response to thermal stress, and thus enable *L. bostrychophila* adults to efficiently deal with ROS induced by thermal stresses. Further, we firstly identified four *LbHsp70* genes and one *LbHsp110* gene from psocid, *L. bostrychophila*. These *LbHsps* are expressed at all development stages with similar expression patterns to some extent. Different expression profiles of *LbHsp70s* and *LbHsp110* in response to high-temperature stresses reveal that *Hsp70* and *Hsp110* genes are functionally involved in the resistance or tolerance to thermal stresses in this pest.

## Figures and Tables

**Table 1:** Primers used for cloning and real-time quantitative PCR (qPCR) in this study.

Experiments	Primer Names	Primer Sequences (5′-3′)	Product Length	Efficiency (%)	*R* ^2^
Full-length confirmation	*LbHsp70-1*F	ATCGTCAGGATCATTTAGTGGCT	2137 bp	-	-
	*LbHsp70-1*R	CGTTTTCGCACAATAAATTCACG			
	*LbHsp70-2*F	CGCGTTGTTAAAATATGGCTGCTC	1541 bp	-	-
	*LbHsp70-2*R	TTACCCACCTCGTCAGGAAATGGA			
	*LbHsp70-3*F	ATAGTTCCTGAGAAATTAACCGAA	2012 bp	-	-
	*LbHsp70-3*R	TCCAGAAGAGGCGTTTAATCAACT			
	*LbHsp70-4*F	TTAAAATATGATCCGTTATAGGAT	1996 bp	-	-
	*LbHsp70-4*R	TCACCCTAATTTCAGTCTTTACAA			
	*LbHsp110-1*F	GTGTGGGTGTTTTAATTTTGTTAT	2532 bp	-	-
	*LbHsp110-1*R	AACGTTATTTATGATTTATTCTGT			
qPCR	q *LbHsp70-1*F	GGGAGAAGATGCCGATCCAG	113 bp	107.5	0.991
	q *LbHsp70-1*R	AACCTTGGTTTTCGCTTGCC			
	q *LbHsp70-2*F	GAAGCTGCTGTTGGTGGAAA	148 bp	105.8	0.981
	q *LbHsp70-2*R	CTGCTGCACATGGTTCATCA			
	q *LbHsp70-3*F	TTGGGTGGAGAGGACTTCGA	163 bp	105.1	0.995
	q *LbHsp70-3*R	TGCTGGCTTGAGTTGACGAT			
	q *LbHsp70-4*F	GGGAAAGAACCGAGTCGAGG	255 bp	103.4	0.993
	q *LbHsp70-4*R	GACTTGGATGGTGACGGTGT			
	q *LbHsp110-1*F	GGGCAGGAGGAATCGAAACA	102 bp	109.7	0.995
	q *LbHsp110-1*R	CGGCAGCTACGCCCATTATA			
	β-actin-F	CACGGTATCGTCACCAACTG	207 bp	98.4	0.998
	β-actin-R	AGACAATACGGCTTGGATGG			

**Table 2:** Malondialdehyde (MDA) concentration (±standard error (SE)) (nmol mg^−1^ protein) of *Liposcelis bostrychophila* exposed to heat shock stresses.

Temperature (°C)	MDA Concentration (nmol mg^−1^ Protein)		
	2 h	4 h	8 h
27.5	0.795 ± 0.087 a ^1^	0.795 ± 0.087 a	0.795 ± 0.087 a
37.5	1.237 ± 0.147 a	2.116 ± 0.295 b	1.711 ± 0.085 a
40	1.022 ± 0.107 a	2.263 ± 0.376 b	1.170 ± 0.498 a
42.5	7.763 ± 0.332 b	17.50 ± 0.263 c	5.720 ± 1.088 b
45	0.846 ± 0.159 a	2.943 ± 0.289 b	NA

^1^ Means within a column followed by the different letters are significantly different (*p* < 0.05) in analysis of variance (ANOVA). NA indicates that no data are available, because all insects died when the treatment last 8 h at 45 °C.

## Supplementary Materials

The following are available online at https://www.mdpi.com/2075-4450/11/12/839/s1, Figure S1: Multiple amino acid sequence alignments of four Hsp70s from Liposcelis bostrychophila, Figure S2: Multiple alignments of the deduced amino acid sequences of Hsp110s from Liposcelis bostrychophila and other insect species, Table S1: Molecular properties of five Liposcelis *bostrychophila* heat shock protein genes.
