# Supplementary material for: Antioxidant Enzymes and Heat Shock Protein Genes from Liposcelis bostrychophila Are Involved in Stress Defense upon Heat Shock
Source: Insects. 2020 Nov 27;11(12):839. doi: 10.3390/insects11120839 (PMC7759835; doi:10.3390/insects11120839)
Supplement: Supplementary file 1 [file insects-11-00839-s001.pdf]

Table S1. Molecular properties of five *Liposcelis bostrychophila* heat shock protein genes.

Gene	GenBank No.	ORF Length	Protein Length	Molecular Weight	pI
LbHsp70-1	MN599717	2070 bp	690 aa	167.4 kDa	4.97
LbHsp70-2	MN599718	1515 bp	505 aa	125.2 kDa	5.06
LbHsp70-3	MN599719	1971 bp	657 aa	161.2 kDa	4.95
LbHsp70-4	KR349165	1971 bp	657 aa	159.3 kDa	4.95
LbHsp110-1	KR349166	2487 bp	829 aa	201.3 kDa	4.91

```
LbHSP70-1     MLYVARNVGRKALNHTNFTTEKMVLKNVSTLVKKALSPSPYLKYNQIRLKSDTVKGAVIGIDLGTTNSCVAVMEG.KQAKVIENAEGARTTPSVVAFTKD     99
LbHSP70-2     ......................................................MAALFGIHVGNSSASLAIYKDEDRVDIVANDAGERVTPTVVQWT.P     45
LbHSP70-3     ....................................................MAKAPAVGIDLGTTYSCVGVFQH.GKVEIIANDQGNRTTPSYVAFT.D     46
LbHSP70-4     ...........................MIRYRILGLIGCLLAVAWAAKDDKNPDVGTVIGIDLGTTYSCVGVYKN.GRVEIIANDQGNRITPSYVAFTPD     72
Consensus                                                                gi  g                   n  g r tp  v  t

LbHSP70-1     GERLVGMPAKRQAVTNSANTFYATKRLIGRKFEDPEVKKDMKTLSYKVVKASNGD..AWIQGQDGKMYSPSQVGAFVLIKMKETAEAYLN.TSVKNAVVT    196
LbHSP70-2     TNCIVGRAAQIEQFRYTTCTVIRNKQLLNENISQSEFEGLKSQIAYKVNKTESNVKYELLFEGKQHHVSPREIVSLIFKKIYGIAEAAVGGNKEMNAVLC    145
LbHSP70-3     TERLIGDAAKNQVAMNPNNTIFDAKRLIGRRFEDATVQSDMKHWPFTVISDGGKPKIQVEYKGETKTFFPEEISSMVLTKMKETAEAYLG.KTVTNAVIT    145
LbHSP70-4     GERLIGDAAKNQLTTNPENTIFDAKRLIGREWTDSTVQHDIKYFPFKVKEKNSKPHIEVQTSQGAKIFAPEEISAMVLGKMKETAEAYLG.KKVTHAVVT    171
Consensus          g  a          t    k l                    v                     p         k    aea         av

LbHSP70-1     VPAYFNDSQRQATKDAGQIAGLNVLRVINEPTAAALAYGMDKS....EDKIIAVYDLGGGTFDISILEIQKGVFEVKSTNGDTFLGGEDFDNLLVNFLVN    292
LbHSP70-2     VPLYFSNKSIELIKDIATKVGFKILQVVDEPCAAALGYNLAVE.PEESLSYCLIYRVGGFSIDATILLIQDGMFSVLGYVHKHNLGGSLFTEKLSEFLAA    244
LbHSP70-3     VPAYFNDSQRQATKDAGAIAGLNVLRIINEPTAAAIAYGLDKKTASTGERNVLIFDLGGGTFDVSILTIEDGIFEVKSTAGDTHLGGEDFDNRMVNHFVQ    245
LbHSP70-4     VPAYFNDAQRQATKDAGTISGLVVMRIINEPTAAAIAYGLDKR...EGEKNVLVFDLGGGTFDVSLLTIENGVFEVVSTNGDTHLGGEDFDQRVMDHFIK    268
Consensus     vp yf        kd      g       ep aaa   y                  gg    d   l i  g f v       lgg   f

LbHSP70-1     EFKKEQGIDITKDPMAVQRLKEAAEKAKIELSSSLQTDINLPYLTVDSSGPKHMNLKLSRSKFESLVDSLIKKTVAPCQKALQDADVKKSDIGEVLLVGG    392
LbHSP70-2     EFKRQYR.TTDITERGKFKLLAAAENVKQILSIQNVAQCSVESIGGGYD....LSCSVSRARFENIMLPFIPEMLSPITEVLNQTGLSVNQINKVIGCGG    339
LbHSP70-3     EFKRKYKKDLTSNKRALRRLRTACERAKRTLSSSTQASIEIDSLFEGVD....FYTSITRARFEELNADLFRSTMEPVEKSLRDAKMDKSQIHDIVLVGG    341
LbHSP70-4     LYKKKKGKDIRKDNRAVQKLRREVEKAKRALSASHQVRIEIESFFEGDD....FSETLTRAKFEELNMDLFRSTMKPVQKVLEDADMNKKDVDEIVLVGG    364
Consensus       k                l    e  k  ls                           r  fe            p    l                gg

LbHSP70-1     MTRMPKVQQTVQEIFG.RQPSKSVNPDEAVAVGAAVQGGVLAGD........VTDVLLLDVTPLSLGIETLGGVFTRLISRNTTIPTKKSQVFSTAADGQ    483
LbHSP70-2     SLKIPKLQESICSLFPSAEALINIAPDELIALGAAKQAGYCIKYENLPESLSIDTPALSNSLSLTVRQGNSNEEESYLILKNSSVPLRR.RYNVLSAENS    438
LbHSP70-3     STRIPKVQKLLQDFFNGKELNKSINPDEAVAYGAAVQAAILHGDK....SEEVQDLLLLDVTPLSLGIETAGGVMTTLIKRNTTIPTKQTQTFTTYSDNQ    437
LbHSP70-4     STRIPKIQQLVKEFFGGKEPSRGINPDEAVAYGAAVQAGVLTGE......QDTDGIVLLDVNPLTMGIETVGGVMTKLIPRNTVIPTKKSQIFSTASDNQ    458
Consensus         pk q      f          pde  a gaa q                    l     l             li  n   p

LbHSP70-1     TQVDIKVHQGEREMASDNKLLGQFTLVGIPPAPRGVPQIEVTFDIDANGIVHVSARDKGTGKEQQIVIQSS.GGLSKDEIENMVKNAEQYAQADKVKKDR    582
LbHSP70-2     LSVDVDIPE......KNNLNLGSVSLNDLEPDS....TVIIELDINKDGKLLLNLVNESSSRKNCLKFDLNEMSIS........................    504
LbHSP70-3     PGVLIQVYEGERAMTKDNNLLGKFELTGIPPAPRGVPQVEVTFDIDANGILNVTACEKSTGKENKITITNDKGRLSKEEIERMVNEAEKYKAEDEKQKAT    537
LbHSP70-4     HTVTIQVYEGERPMTKDNHLLGKFDLTGIPPAPRGVPQIEVTFEIDANGILQVSAEDKGTGNKEKIVITNDQNRLTPDDIERMIKDAEKFADEDKRLKEK    558
Consensus       v              n  lg   l    p             i   g

LbHSP70-1     VEARNSAEGIIHQTESKMDE...FKSQLPQEECDKLRTQITELRSLLDKGEDADPEEIRKSSTSLQQASLKLFEMAYRKMASENQGSP..........GS    669
LbHSP70-2     ....................................................................................................    504
LbHSP70-3     ISAKNALESYCFNMKSTIED.EKLKDKISETEKQTIMDKCNDIIKWLDANQLAEAEEYEHKQKELEAICNPIITKLYQGAGGMPGGMPGGMPGGFPGAGG    636
LbHSP70-4     VESRNDLESYAYSLKNQLGDKEKLGAKVSDEDKAKMEEAIDEKIKWLDQNPDADVEEYKKQKKELEDVTQPIIAKLYSSTGGAP................    642
Consensus

LbHSP70-1     SSSEQTEQQEPKEEKKEEKN                                                                                689
LbHSP70-2     ....................                                                                                504
LbHSP70-3     AAPGGAPGAGGTGPTIEEVD                                                                                656
LbHSP70-4     .....PPSAGDDDDLKDEL.                                                                                656
Consensus
```

Figure S1. Multiple amino acid sequence alignments of four *Hsp70s* from *Liposcelis bostrychophila*. Three signature sequences of the Hsp70 family are indicated by grey shading. The putative ATP-GTP binding site is colored with yellow shading. The potential non-organelle eukaryotic consensus motif and putative bipartite nuclear location signal are in green and purple shading, respectively.

```
CsHSP110      MAAMSVIGIDFGNESCYVAVARAGGIETIANDYSLRATPSCVALSGKNRILGVAAKNQMVTNMKNTIHGFKRFLGRKYNDPHVQQELKYLPFRVVQQPNG   100
LbHSP110-1    ...MSVIGIDFGNESCYIAVARAGGIETIANDYSLRATPSCVAFSDRNRIMGVAAANQTVTNMKNTIVGFKRLLGRKFNDPYVQQEMQFLPYAVVPNQDG    97
PhHSP105      ...MSVIGIDFGNESCYIAVARAGGIETIANDYSLRATPSCVAFSDRNRIMGVAAANKRVTNMKNTIVEFKRLLGRKYNDPYVQHELQYLPFSVVPNQDG    97
Consensus        msvigidfgnescy avaraggietiandyslratpscva s  nri gvaa n  vtnmknti  fkr lgrk ndp vq e   lp  vv    g

CsHSP110      SIGVKVNYLDEEHTFSPEQITAMLFTKLKEVSENALKTKVNDCVISVPSFFTNAERKALLDAASIAGLNVLRLMNETTATALAYGIYKQDLPAPEEKPRN   200
LbHSP110-1    SLGIKVHYMNEEHVFTPEQITAMLFTKLKEIAESALKTKVNDCVISVPSFYTQAERKALLDAAHIAGLNVLRLFNETTSTALAYGIYKQDLPNAEEKPRN   197
PhHSP105      SLGIKVNYLNEEHIFTPEQITAMLFTKLKEIAENALRTKVNDCVISVPSFYTQAERKSLLDAAHIAGLNVLRLFNETTSTALAYGIYKQDLPNAEEKPRN   197
Consensus     s g kv y  eeh f peqitamlftklke  e al tkvndcvisvpsf t aerk lldaa iaglnvlrl nett talaygiykqdlp  eekprn

CsHSP110      VVFVDCGHASLQVFACAFHKGKLKMLASASDPNLGGRDIDLILAEHFCTDFQSRYHIDPHSNHRAFLRLTSEVEKLKKQMSANSTKLPMNIECFMDDKDV   300
LbHSP110-1    VVFVDCGHSSLQVFAAAFNKGKLKMLASASDASLGGRDFDRILAEHFSKEFVSKYKIDPRKNARAYLRLTTEVEKLKKQMSANSTKLPLNIECFMEDKDV   297
PhHSP105      VVFVDCGHSSLQVFAAAFNKGKLKMLASAS.SSIGGRDIDRILAEHFSKEFVSKYKIDPRKNARAYLRLMTEVGKLKKQMSANSTKLPLNIECFMDDKDV   296
Consensus     vvfvdcgh slqvfa af kgklkmlasas    ggrd d ilaehf   f s y idp  n ra lrl  ev klkkqmsanstklp niecfm dkdv

CsHSP110      HGDMKRADMEQLCGHLFKRVEKTLRKCLEDSKLHLEEIHAVEIVGGSSRIPAIKHVIEVVFGKQPSTTLNQDEAVSRGCALQCAMLSPAIRVREFSITDI   400
LbHSP110-1    HSGLQRSDMEGLCSGLIARVERTLRKCLADSKLSLDDIYAVEIVGGSTRIPSIKNLIEDIFKKHPSTTLNQDEAVARGCALQCAALSPAIRVREFSVTDI   397
PhHSP105      HSDMQRSDMEELCAGLFQKVEKTLTKCLKDSNLSLEEIYAVEIVGGSTRIPAIKNLIEEVFKKQPSTTLNQDEAVARGCALQCAALSPAIRVREFSVADI   396
Consensus     h    r dme lc  l   ve tl kcl ds l l  i aveivggs rip ik  ie  f k psttlnqdeav rgcalqca lspairvrefs  di

CsHSP110      QNYPIKLVWDASMGEDG.EMEVFSQNHAVPFSKMLTFFRKEPFSMKAFYAGNIPYPDPYIGQFVVKDVKPSPEGESVKVKVKVRVNLHGILTISSASLVL   499
LbHSP110-1    QNFPVVLKWDAAPGEEGGEMEIFSANHAVPFSKLLTFYRREPFSVQAFYSGQTPYPDPYIGRFTIKDVHPTPENESAKVKVKVRINLHGILTVAGATLTE   497
PhHSP105      QNYPVTLKWDAYPGEEEGEMEIFTANHAVPFSKLLTFYRKEPFSVHAFYSHPTSYPDSYIGKFTIKDVRPTVDNEPATVKVKVRINLHGILTVAGATLTE   496
Consensus     qn p  l wda  ge   eme f  nhavpfsk ltf r epfs  afy     ypd yig f  kdv p    e   vkvkvr nlhgilt   a l

CsHSP110      KQDASEQDTNEQDNAGPEAMDTEAQKNE..VNGSANQ.QQQGQTENSQ..ANHIGNEDDTENQTASEKKDG.TRKKKQLVKTVELPIEAYTHGYSQAELN   593
LbHSP110-1    KLTQAEIDAEANKEAMETDTNTEQPVNN..VENGEEK.EQTNEEEMDQQPAQEQSNQQNQQQQQNQQGSDKKEKKKKQLVKNVDLSIESETHGLSQLQLN   594
PhHSP105      KLTQAEIEAESKESMELDSNSQEQQDQNNWMENGEEQGKDKDQGNEDENDQSKDQQQSQSQQQQQQQSADKKEKKKKSHVKKIDLNIDTETHGLTKLQLD   596
Consensus     k    e                e                                       q      d    kkk vk   l i   thg     l

CsHSP110      NYIELECKMVASDRQEKERIDARNALEEYVYEVRGKLSSEEELATFVSENDRNSLCRQLDNMENWLYEEGEECNRQIYVDKLNVLKNLGEPIKTRRLEFE   693
LbHSP110-1    SYYEFEGKMMANDKQEKERVDARNALEEYVYDLRGKLTDDSQLANYVTDQDREGLSSQLNDMENWLYEEGEDCQRQVYNDKLDQLKKLGEPIKNRKIEAD   694
PhHSP105      SFYEFECKMIANDRQEKERADARNAMEEYVYDLRGKLGDEAQLASFVNDADRDSLMSQLNDMESWLYEEGEDCNRQIYIDKLDQLKKLGEPIKNRKIEAD   696
Consensus        e e km a d qeker darna eeyvy  rgkl     la  v   dr  l  ql  me wlyeege c rq y dkl  lk lgepik r  e

CsHSP110      LRPNALEELACVLQLTRKAVDQYRAGDERYSHLEESDIQKVQQTAEQTHRWLEEKRAALAGTPRTQNPPVTVAQIRQEKQNFENVVNPILTKPKPKADPP   793
LbHSP110-1    IRPTVFEDLARSIQQSYKIIDQYRNKDERYNHIPEEDILKAQQETERVGKWLDEKRAKAQSTPKYENPPVTVAQIRQEKQNFENIVAPIFNKPKPKPKNE   794
PhHSP105      IRPTVIEDLARSIQQSYKIIDQYNNNDERYNHIPEGDINKALQETQKVEKWLDEKRMKIQSTPKYENPVVTVAQIRQEKQAFENLVAPIFNKPKPKPKTE   796
Consensus      rp   e la   q   k  dqy   dery h  e di k  q       wl ekr     tp   np vtvaqirqekq fen v pi  kpkpk

CsHSP110      PPVNKENGNTAEKKDAQEDDSPKQEQQSDENMDVE...                                                                 828
LbHSP110-1    TPPAPAP.EENQKATGDSNQPQGETNNAEEKMETE...                                                                 828
PhHSP105      APPPTAAPPSSEDANNKATGDSVEPNNFEEPMETDSFI                                                                 834
Consensus      p                           e m
```

Figure S2. Multiple alignments of the deduced amino acid sequences of *Hsp110s* from *Liposcelis bostrychophila* and other insect species. The amino acid sequences constituting a single ATP-binding motif are in green color. The amino acid letters with red color indicate HSPA4 like nucleotide-binding domain, and the letters in blue color indicate periplasmic substrate-binding domain. Cs: *Cryptotermes secundus*, Ph: *Pediculus humanus corporis.*
